# Dataset of Pakistan Sign Language and Automatic Recognition of Hand Configuration of Urdu Alphabet through Machine Learning

**DOI:** 10.1016/j.dib.2021.107021

**Published:** 2021-04-02

**Authors:** Ali Imran, Abdul Razzaq, Irfan Ahmad Baig, Aamir Hussain, Sharaiz Shahid, Tausif-ur Rehman

**Affiliations:** a Department of Computer Science, Pakistan; b Department of Agribusiness and Applied Economics, Muhammad Nawaz Sharif University of Agriculture, Multan, Pakistan

**Keywords:** Pakistan sign language, Hand configuration, Machine learning, Deaf people communication, Mobile app

## Abstract

Social correspondence is one of the most significant columns that the public dependent on. Notably, language is the best way to communicate and associate with one another both verbally and nonverbally. There is a persistent communication gap among deaf and non-deaf communities because non-deaf people have less understanding of sign languages. Every region/country has its sign language. In Pakistan, the sign language of Urdu is a visual gesture language that is being used for communication among deaf peoples. However, the dataset of Pakistan Sign Language (PSL) is not available publicly. The dataset of PSL has been generated by acquiring images of different hand configurations through a webcam. In this work, 40 images of each hand configuration with multiple orientations have been captured. In addition, we developed, an interactive android mobile application based on machine learning that minimized the communication barrier between the deaf and non-deaf communities by using the PSL dataset. The android application recognizes the Urdu alphabet from input hand configuration.

## Specifications Table

**Table:** 

Subject	Sign Language and Machine learning based translation of Urdu Alphabets
Specific Subject area	Generation of Pakistan Sign Language (PSL) dataset and automatic recognition of Urdu character of the input symbol through Mobile App.
Type of data	Images files.
How Data were acquired	Multi-orientations and shape Images (Hand configuration) are captured through webcam.
Data format	Raw Analyzed.
Parameters for data collection	All special hand configuration of the Urdu language alphabets are captured with different orientation and shapes of both hand and fingers [15]
Description of data collection.	There are 37 letters in the Urdu alphabet. Forty (40) images of each alphabet have captured. Each class of image are labeled with Urdu alphabet. The proposed dataset contained total 1480 images.
Data source location	Institution: Department of Computer Science, MNS-University of Agriculture Multan, Punjab, Pakistan.
Data Accessibility	Direct URL of Data https://data.mendeley.com/datasets/y9svrbh27n/1
Code Accessibility	Direct URL of Code https://data.mendeley.com/datasets/26kndg8xsw/2

## Value of the Data

• The dataset based on static hand configuration that could be used as a mean of communication between the deaf and non-deaf communities.
• The proposed dataset could be used for developing a cost-effective, easy to use and interactive application for translating the Urdu language to overcome the communication gap between deaf and non-deaf people.
• The proposed dataset consists of static poses of the sign alphabets that could be used to spell out words and sentences.
• Sign Language dataset and its recognition is an active area of research in the field of gesture recognition, robotics, gesture-based authentication.
• The proposed PSL dataset is a valuable resource for hearing-impaired students, their families, and the special education system in Pakistan.

## 1 Data Description

Sign language is a way of communication among deaf communities. The sign language consists of dynamic or static hand configuration [2], [3]. Sign alphabets are based on static hand poses which represent the letters of the alphabets and it uses gestures besides verbal communication [11], [12], [13], [14]. However, in the existing system, deaf people face a major problem to communicate with non-deaf people through sign language because non-deaf people do not understand it. The advancement in computer vision enables us to produce some complex models that can recognize the sign, hand configuration, and translate it into text and voice [6]. These models require a dataset to get trained. However, the dataset of Pakistan sign language (Urdu) is not available publicly.

In this work, we developed a Pakistan sign language dataset for the Urdu alphabets recognition. There are 37 alphabets in the Urdu language, in our developed dataset total of 40 images for each alphabet are captured with different orientation of hand and fingers through webcam. The alphabets of Urdu sign language are categories baesed on the shape and orientations of both hand and fingers as described in [15]. Total 1480 images are acquired and with these images, 37 classes are created. Each class of images is labeled as one alphabet of Urdu language. The developed dataset will help the researchers in the field of sign recognition and translation to develop communication systems for deaf people in Pakistan. In addition, we developed an interactive machine learning-based android app with the developed dataset. The purpose of the developed application is to recognize the hand configuration sign through a mobile camera. The model for the PSL hand configuration recognition application is trained through a support vector machine (SVM). The trained system automatically recognizes the static hand configuration sign for the Urdu alphabet.

## 2 Experimental Design, Materials and Methods

**Fig. 1:**
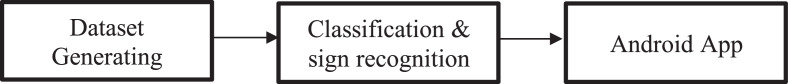
System phases.

The proposed system is based on three phases Fig. 1

(1) Dataset Generating.
(2) Sign recognition and classification through machine learning.
(3) Developing Android app for end user.

### 2.1 Generating dataset

**Fig. 2:**
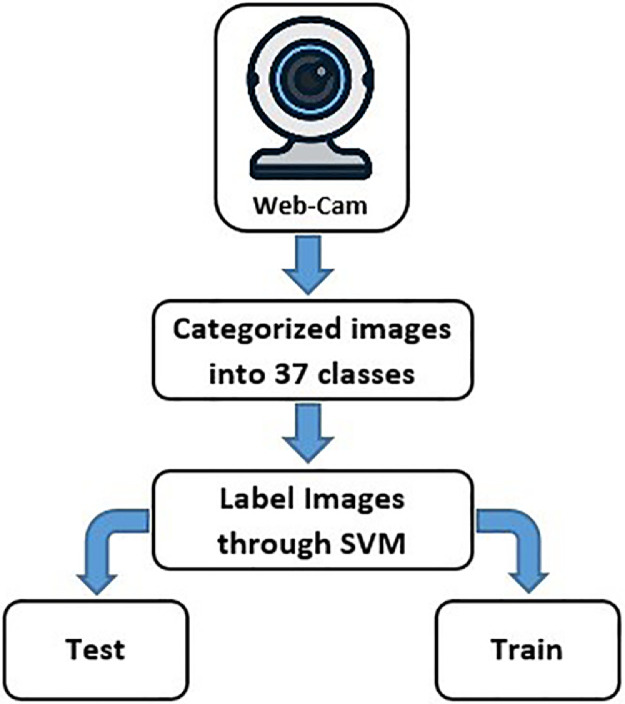
PSL data generation.

The sign language dataset has been generated followed three steps as illustrated in Fig. 2. The first step was to capture the images of signs using a webcam, forty images of each sign were captured with different poses and orientations of hand and fingers. In a second step, the captured images are categorized into 37 classes each class contained forty images of one sign with different orientations. In the third step after the categorization of images, we assign the 37 alphabets of Urdu language to each class accordingly. In the last, the created image dataset has been annotated through a classification system, Support vector machine (SVM), and stored in XML file format. Further, the annotated dataset was divided into two subfolders train and test with a 70 and 30% ratio, respectively.

### 2.2 Sign recognition through SVM

Recognition of sign image is performed through machine learning algorithm namely Support Vector Machine (SVM). The sign recognition has accomplished through segmentation, detection, and recognition of the input symbol.

#### 2.2.1 Segmentation

**Fig. 3:**
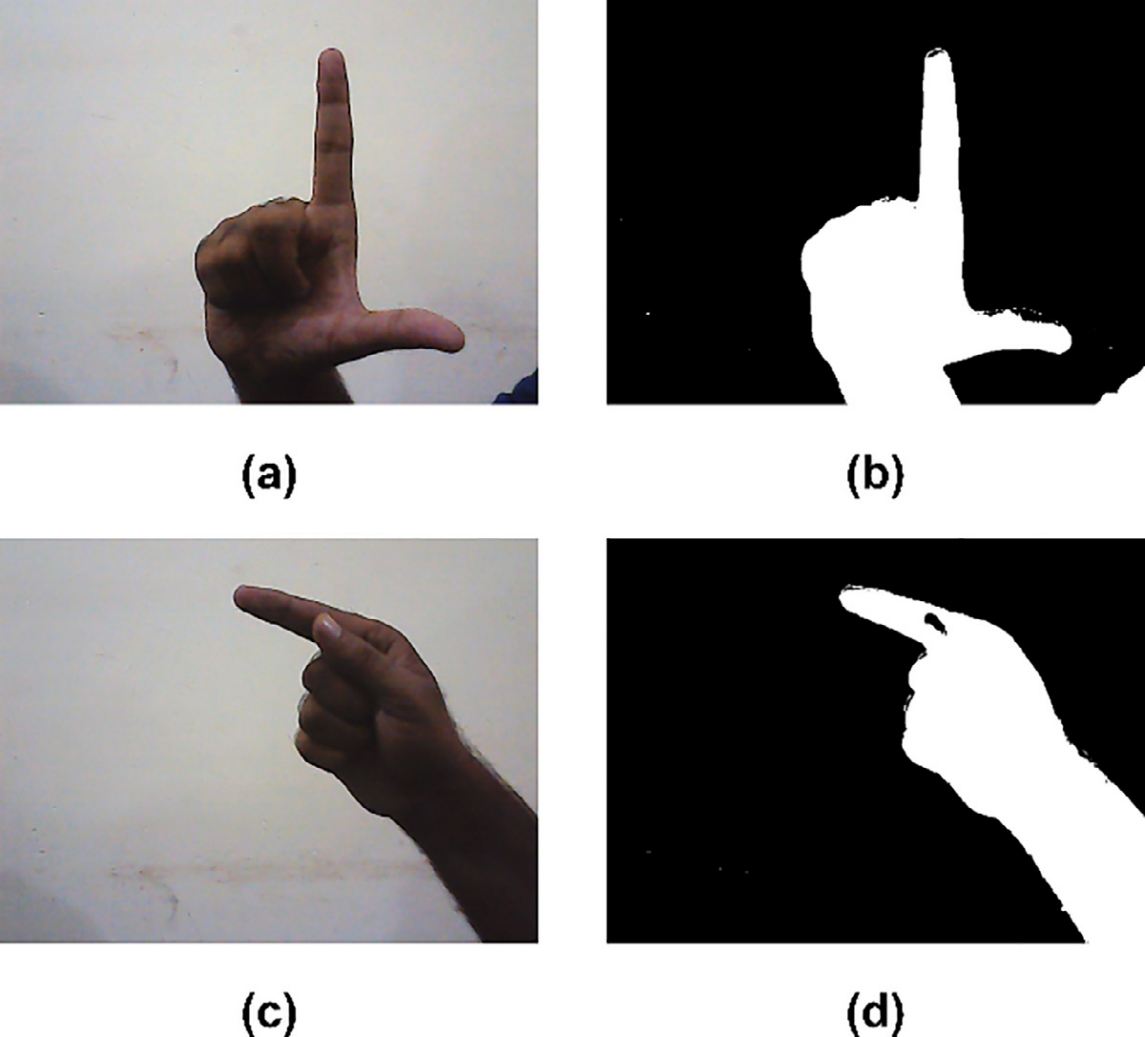
An example of segmentation the original and segmented image respectively.

In hand configuration recognition, segmentation is an important step to get a successful result. The purpose of segmentation is to isolate candidate hand signs from the background. From the input image, the colored information is noted as a candidate object. The hard task in hand configuration segmentation is to define the changes in different orientations of hand signs. In RGB images it's a very difficult task to find the correct thresholds value for the sensitivity of light and co-relation between the three components in an empirical way. The HIS color has two component colors, one is hue and the second is saturation and the important thing is that it is closely related to human perception. The hue expresses the dominant color and saturation shows the purity of color with high and low color values of white. In the proposed system saturation and hue components of HIS space are good enough to isolate hand configurations with fixed threshold under a broad range of light conditions. From hue and saturation work the values of the threshold has fixed. The Hue and Saturation components do not have information to segment white objects. An achromatic decomposition has implemented for analyzing the ratios between the RGB components [7]. The result of the segmentation is illustrated in Fig. 3 which include only the interest masks.

#### 2.2.2 Detection

The detection process is divided into two sub-steps: at first, a shape classification in which the identification of orientations and sign are compares with the signature of the theoretical shapes of an equilateral shape. To make the algorithm constant to object rotation the comparison of the signature signal has performed through the FFT rather than of signature itself. For object scaling the algorithm is constant through the normalization of the energy of the signature. The advantage of the implemented algorithm is its invariance which is used for object translation, scaling, rotations, and robustness to camera projection [5]. The output is the blob list which is obtained from the segmentation step and updated with its estimated shape. Secondly, the localization of the hand shape achieves by implementing homography that allows the system to place the hand configuration in the reference position.

#### 2.2.3 Sign recognition

When the candidate blob has been selected as a possible hand configuration, the objective of the recognition stage is to recognize the sign i.e. (which sign belongs to which alphabets). In the proposed system sign recognition has been carried out through SVM in which the input vector is normalized. We use binarization as the first approach however the results we get are sensitively worst. In the real-world, the hand configuration is affected by light changes, shadows, and occlusions which make the simple thresholding incite important confusions at the recognition stage [10]. However, it is hard to find adaptive thresholding like [9] algorithm which helps to introduce no distortion in any sign sample due to lack of information of the original level of intensity. In order to remove feature vectors, the pixels that cover the area of the hand configuration are calculated with the mask, as described in [8]. The problem which is under consideration is that a multi-class one with samples that do not belong to any sign. The noise samples come from the detection and segmentation stage. For the recognition process, the training and testing are performed according to the shape of each signature of the hand configuration [1]. Therefore, every hand configuration is only compared with those signs which have the same shape properties as the hand blob to be recognized. The extension of the SVM learning method, in this case, is performed by the one against all strategy [4] and the number of classes *N* needed is equal to the number belong to the case considered. On the other hand, when two or more than two classifiers produce a positive result, the one with the highest output is taken the radial basis function (RBF) kernel, and other kernels such as polynomial or sigmoidal ones gave worse results.

$$N\left(x,y\right)=x{\;}^{-\gamma \sum_{i=1}^{n}{\left(x_{i}-y_{i}\right)}^{2}}$$

**Fig. 4:**
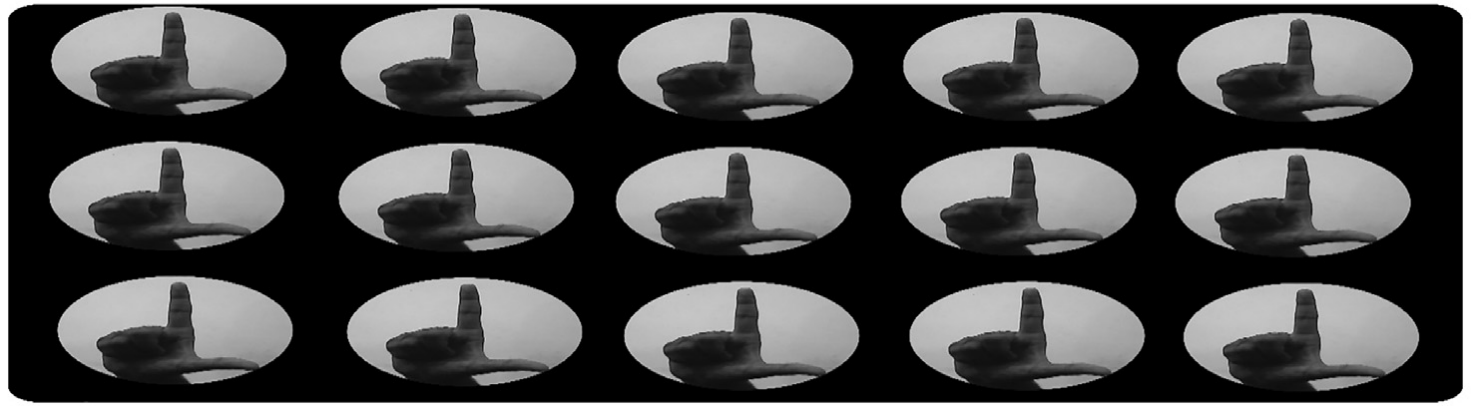
Positive support vectors for ‘ل’ alphabet.

**Fig. 5:**
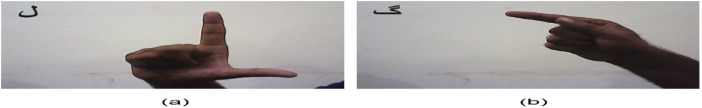
Results of a sign recognition output.

Some vector patterns of the whole set define the decision hyperplane. These vectors are called support vectors. In Fig. 4 the positive vectors are shown that define the binary decision region for the Urdu alphabet ‘ل’, due to the size normalization of each blob, the method is invariant to scale changes. In Fig. 5 the sign recognition results are illustrated. Where all identified objects are strained over the image with their particular shape.

### 2.3 Android application development

**Fig. 6:**
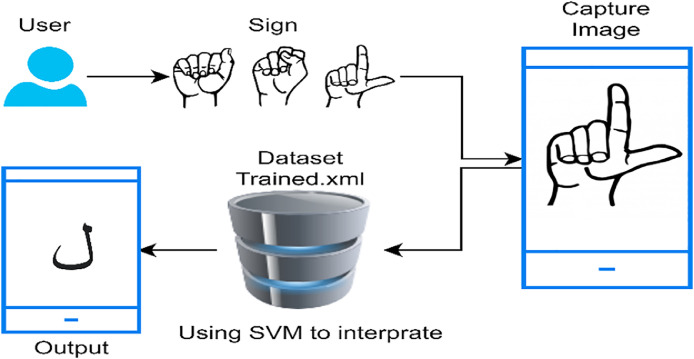
Workflow of android application.

An android application has developed and trained on developed PSL datasets for end users. The open-cv library has used to develop a system for the app. Open-cv supports the SVM algorithm build. The trained algorithm recognizes the PSL and translates it into the Urdu alphabets. The workflow of the application consists of the following steps Fig. 6

• Takes input image of hand sign from the camera.
• Use trained SVM Interpreter.
• Display the result of the Urdu alphabet of the input sign.

This paper proposed a dataset of sign language and android-based communication systems which is a hand configuration acknowledgment framework for Pakistan Sign Language (PSL). The dataset of the PSL is available publicly on the cloud and could be used by anyone and the framework makes an interpretation of the communication through sign-to-text translation by using a mobile camera. The system accomplished 80–90% accuracy on different tests in various light conditions tabulated in Table 2. We tested our model on different hand configurations. To evaluate the performance of the proposed method, several quantitative indices were calculated which include accuracy, precision, recall, and F1 score. The parameters that are widely used to evaluate the performance of the models are defined as follows. Table 1

(a) Accuracy

**Table 1:** PSL hand configuration & their corresponding urdu alphabet and name of the folder in PSL dataset

**Tabel 2:** Accuracy of the android app in different experiments

**Fig. 7:**
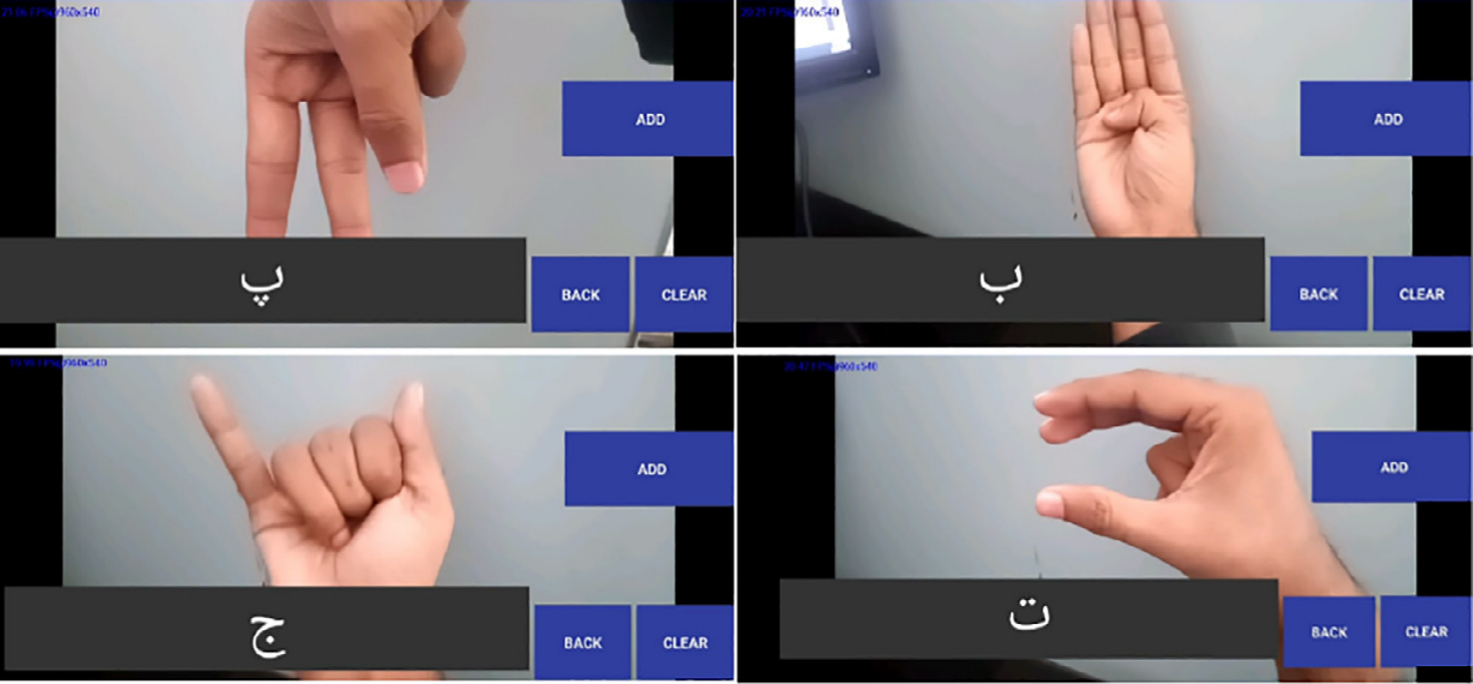
Android application GUI with output.

The accuracy of classifier is calculated as:

$$\text{AUC}=\frac{\text{True}\;\text{Positive}+\text{True}\;\text{Negitive}}{\text{True}\;\text{Positive}+\text{True}\;\text{Negitive}+\text{Flase}\;\text{Positive}+\text{False}\;\text{Negitive}}$$

(b) Precision
  $$\;\text{Precision}=\frac{\text{True}\;\text{Positive}\;}{\text{True}\;\text{Positive}+\text{False}\;\text{Positive}}$$
(c) Recall
  $$\text{Recall}=\frac{\text{True}\;\text{Positive}}{\text{True}\;\text{Positive}+\text{False}\;\text{Negitive}}$$
(d) F1 Score
  $$F1\;\text{score}=2\times \frac{\text{Precision}\times \;\text{Recall}}{\text{Precision}+\;\text{Recall}}$$

## 3 Android app GUI

The android ecosystem is diverse, and this is the age of smartphones. In Pakistan, 161.183 million people are using android phones. The android phones are user-friendly and easy to use however, the existing system for PSL translation is only a desktop and web-based application. The proposed system developed an interactive and user-friendly android application that captured signs as input and translate the sign into Urdu alphabets. The developed application helps the deaf peoples in Pakistan and be used in evaluating the developed dataset. Fig. 7 shows the GUI of the android application.

## CRediT Author Statement

All authors contributed equally in this work.

## Funding Statement

This esearch received no specific grant from any funding agency in the public, private sectors.

## Declaration of Competing Interest

The authors have no conflicts of interest to declare
